# DisProt in 2024: improving function annotation of intrinsically disordered proteins

**DOI:** 10.1093/nar/gkad928

**Published:** 2023-10-30

**Authors:** Maria Cristina Aspromonte, Maria Victoria Nugnes, Federica Quaglia, Adel Bouharoua, DisProt Consortium, Vasileios Sagris, Vasilis J Promponas, Anastasia Chasapi, Erzsébet Fichó, Galo E Balatti, Gustavo Parisi, Martín González Buitrón, Gabor Erdos, Matyas Pajkos, Zsuzsanna Dosztányi, Laszlo Dobson, Alessio Del Conte, Damiano Clementel, Edoardo Salladini, Emanuela Leonardi, Fatemeh Kordevani, Hamidreza Ghafouri, Luiggi G Tenorio Ku, Alexander Miguel Monzon, Carlo Ferrari, Zsófia Kálmán, Juliet F Nilsson, Jaime Santos, Carlos Pintado-Grima, Salvador Ventura, Veronika Ács, Rita Pancsa, Mariane Goncalves Kulik, Miguel A Andrade-Navarro, Pedro José Barbosa Pereira, Sonia Longhi, Philippe Le Mercier, Julian Bergier, Peter Tompa, Tamas Lazar, Silvio C E Tosatto, Damiano Piovesan

**Affiliations:** Department of Biomedical Sciences, University of Padova, Padova, Italy; Department of Biomedical Sciences, University of Padova, Padova, Italy; Department of Biomedical Sciences, University of Padova, Padova, Italy; Institute of Biomembranes, Bioenergetics and Molecular Biotechnologies, National Research Council (CNR-IBIOM), Bari, Italy; Department of Biomedical Sciences, University of Padova, Padova, Italy; Department of Biomedical Sciences, University of Padova, Padova, Italy; Department of Biomedical Sciences, University of Padova, Padova, Italy

## Abstract

DisProt (URL: https://disprot.org) is the gold standard database for intrinsically disordered proteins and regions, providing valuable information about their functions. The latest version of DisProt brings significant advancements, including a broader representation of functions and an enhanced curation process. These improvements aim to increase both the quality of annotations and their coverage at the sequence level. Higher coverage has been achieved by adopting additional evidence codes. Quality of annotations has been improved by systematically applying Minimum Information About Disorder Experiments (MIADE) principles and reporting all the details of the experimental setup that could potentially influence the structural state of a protein. The DisProt database now includes new thematic datasets and has expanded the adoption of Gene Ontology terms, resulting in an extensive functional repertoire which is automatically propagated to UniProtKB. Finally, we show that DisProt's curated annotations strongly correlate with disorder predictions inferred from AlphaFold2 pLDDT (predicted Local Distance Difference Test) confidence scores. This comparison highlights the utility of DisProt in explaining apparent uncertainty of certain well-defined predicted structures, which often correspond to folding-upon-binding fragments. Overall, DisProt serves as a comprehensive resource, combining experimental evidence of disorder information to enhance our understanding of intrinsically disordered proteins and their functional implications.

## Graphical Abstract

**Graphical Abstract:**
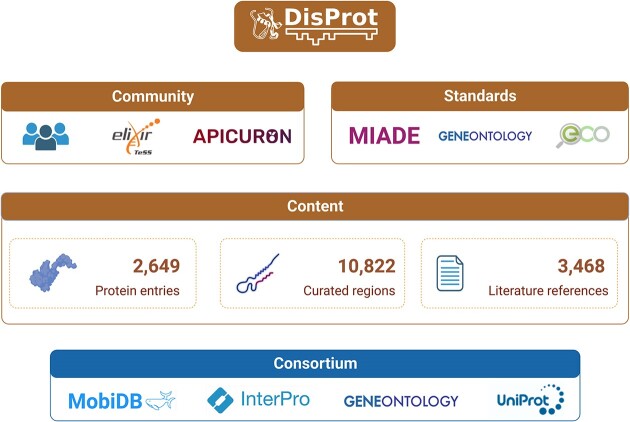

## Introduction

The recent breakthrough in structure prediction achieved by AlphaFold2 (1) has revolutionized biological research, providing reliable putative structures for proteins and regions that previously lacked three-dimensional structures (2). However, the large-scale structural landscape provided by AlphaFold2 confirms that structural disorder may be more prevalent in proteomes than previously thought, opening up new avenues for studying the less-explored regions of the proteome (3).

Intrinsically disordered proteins (IDPs), which exhibit an ensemble of heterogeneous structures with diverse properties and functions (4), are important in cell physiology but also play a central role in diseases and are underexploited targets for drug development (5,6). However, their characterization poses significant challenges at both the experimental and computational levels (7). A fundamental problem is that disorder cannot be simply described as a static binary state; instead, it can be better represented by an ensemble of heterogeneous structures with diverse properties and functions that are often context-dependent. These properties become observable only under specific conditions, such as pH, localization, binding, and post-translational modifications (8).

To expand our knowledge and develop reliable models for dynamic and condition-dependent molecular systems, it is crucial, now more than ever, to accumulate standardized and accessible experimental evidence for IDPs. The DisProt database has been serving this purpose since its initial release in 2005 (9). It extracts knowledge from the literature and transforms it into machine-readable database records through the expertise of a dedicated team of biocurators, whose work is recognized and encouraged thanks to the connection of DisProt with the APICURON platform (10).

The database has been continuously updated and refined to capture structural and functional information from IDP experiments. The systematic adoption of *Minimum Information About Disorder Experiments* (MIADE) guidelines facilitated the exchange of data and enabled filtering of experimental evidence obtained under non-standard conditions. MIADE guidelines define the essential fields required to establish an unambiguous conclusion based on experimental observations. Leveraging the quality and complementary nature of the accumulated evidence, DisProt has been utilized as the reference dataset for the *Critical Assessment of Protein Intrinsic Disorder* prediction (CAID) experiment, which benchmarks ID and binding predictors (7,11).

A significant recent advancement of DisProt is the systematic curation of ID functions. DisProt plays a crucial role in the *Gene Ontology* (GO) Consortium, contributing to both the maintenance of the Gene Ontology and the accumulation of disorder-specific functional annotations (12). Recently, the DisProt function annotations were utilized as the ground truth for a sub-challenge in the *Critical Assessment of protein Function Annotation* (CAFA) challenge (13), however results, at the moment of writing, are not published yet.

DisProt serves as a central IDP resource for several core databases. The positional annotations, specifically disordered sites, are imported into the MobiDB (14) and other IDP databases (15), including PED (16) and FuzDB (17). Function annotations are imported in GO and automatically propagated to UniProtKB.

Compared to the version presented in the previous publication (18) (release 2021_08), the number of DisProt entries increased by 30%. When considering the number of entries with function annotations, the growth is 57%. Also, we introduced 5 new thematic datasets, i.e. collections of proteins where IDPs play a crucial role, and enriched all ambiguous evidence with MIADE attributes.

The DisProt consortium develops training materials for both biocurators and data users (19). Announcements about new features and advancements are communicated through the DisProt blog and the official DisProt account on X (formerly Twitter) social network. The latest version of the DisProt website features a new graphical interface and updated features. With these improvements, DisProt continues to be a primary resource for the structural-molecular biology community in the study of protein disorder. In the following chapters we provide a detailed overview of DisProt advancements achieved since the last publication.

## Progress and new features

### Database growth

The most recent DisProt release 2023_06 showcases significant growth and advancements in the field of structural and functional disorder research. DisProt now includes a total of 2649 proteins representing a growth of 30% compared to the previous publication. There are 10 822 pieces of experimental evidence available in DisProt, which provide insights into structural and functional disorder. These pieces of evidence are mapped to specific sequence regions within the proteins. The growth of annotated regions is about 20% and notably 43% are flagged as validated, meaning a senior curator checked the correctness of the annotation. The number of distinct literature articles used to extract annotations is 3468, showing a 35% increase compared to the previous version. The number of covered species (407 organisms) has increased by approximately 15%. The number of curators involved in maintaining and updating DisProt has also increased by around 15%. DisProt has also witnessed a remarkable expansion in the number of new function annotations. Specifically, 3971 regions in 1297 proteins are currently annotated with functions, more than doubling the previous count. Overall, the DisProt release 2023_06 demonstrates substantial growth in the number of proteins, regions, and literature references, as well as an increase in species coverage and curator participation. The most notable advancements include the rise in validated regions and the doubling of new function annotations, which contribute significantly to our understanding of structural and functional disorder in proteins.

Part of the newly annotated proteins were tagged with a name indicating a specific biological area where IDPs play a crucial role. Groups of tagged proteins are called ‘thematic datasets’ and are meant to improve user accessibility to specific themes. Compared with the previous publication 5 new datasets have been added as shown in Table 1.

**Table 1.** Number of proteins and regions included in the thematic datasets and in the whole DisProt database

Dataset	Proteins	Regions
NDDs-related proteins	312	1212
Viral proteins	210	1314
Cancer-related proteins	146	952
Autophagy-related proteins	101	802
Neglected tropical diseases proteins	101	249
Extracellular matrix proteins*	66	352
Unicellular toxins and antitoxins*	47	294
DisProt total annotations	2649	10 822

NDDs = neurodevelopmental disorders. (*) Datasets already available in the previous publication.

### Annotation quality

In addition to the validation process, where expert curators check the correctness of the annotations collected by other curators, the last DisProt release (2023_06) includes a number of regions that are annotated with Minimum Information About Disorder Experiments (MIADE) descriptors (20). MIADE guidelines define the fundamental fields that are necessary to support an unambiguous conclusion based on experimental observations. These include construct definition, which provides information about tags, labels, mutations and modifications, and the experimental setup, which describes complex experimental samples and parameters in detail. In DisProt, MIADE fields are represented in a standardized way using stable external identifiers and controlled vocabularies (CVs). Table 2 shows the distribution of MIADE annotations in the last DisProt release. MIADE replaces the ‘ambiguity’ tags used in previous DisProt versions, which were used to indicate ambiguity at the experimental level but without providing detailed and standardized information.

**Table 2.** MIADE fields in DisProt

MIADE field	DisProt field	Ontology/CV	Proteins	Regions
Construct alterations	Protein mutation	HGVS nomenclature	77	183
	Protein modification	PSI-MOD ontology	31	89
	Tag	PSI-MI ontology	22	63
	Non-standard amino acid	PSI-MOD ontology	15	23
	Label and dyes	PSI-MI ontology	3	6
Experimental conditions^a^	pH	NCI Thesaurus OBO Edition CV	13	34
	Temperature	NCI Thesaurus OBO Edition CV	4	8
	Pressure	NCI Thesaurus OBO Edition CV	0	0
	Oxidation-reduction potential	NCI Thesaurus OBO Edition CV	0	0
Sample	Interacting small molecule	IDPO ontology	152	356
	Interacting protein	IDPO ontology	134	278
	Interacting nucleic acid	IDPO ontology	12	27
	Interacting lipid	IDPO ontology	8	16
	Interacting antibody	IDPO ontology	8	11
	Interacting membrane	IDPO ontology	5	11
	In-cell experiment	IDPO ontology	3	5

^a^The units of parameters describing experimental conditions are defined in the Measurement Ontology and deviations from the expected value in the experimental parameters are expressed by the following descriptors: ‘within normal range’, ‘increased’, ‘decreased’, ‘not specified’, ‘not relevant’.

### Disorder content

The fraction of disordered residues, or disorder content, is an important indicator that can be used to distinguish fully disordered proteins from structured proteins with a small disordered region. Sometimes knowing the disorder content of a protein is more important than knowing the exact position of disordered regions along the sequence, for example to guide experiments, or it can be directly used as a starting point to generate hypotheses about the function of the protein (21). Therefore, in DisProt we pay attention to capture as much evidence as possible about the structural state of the entire protein. To this end, in the last release we started to use a new set of Evidence and Conclusion Ontology (ECO) (22) terms, which allowed us to extend the range of literature evidence that can be included in DisProt and therefore increase the disorder content at the database level. In the previous publication we described the adoption of a new ECO term to capture obvious cases like poly-glutamate, poly-lysine, etc. which are easily detected by automatic methods but for which experimental validation is often lacking since they are known to be always disordered. In the last version of DisProt we introduced new ECO terms in order to capture authors and curators inferences and statements. In DisProt the most used ECO terms of this category are ‘combinatorial experimental and author inference evidence contained in single publication used in manual assertion’ (ECO:0006218), ‘curator inference used in manual assertion’ (ECO:0000305) and ‘author statement used in manual assertion’ (ECO:0000302).

For example, the disorder content of *SH3 and multiple ankyrin repeat domains protein 3* (DisProt DP02376) was 0.7% and after the inclusion of four author statement evidence (ECO:0000302) became 63.6%. Another example is the *Tegument protein VP16* (DisProt DP02291), which thanks to an ‘curator inference used in manual assertion’ (ECO:0000305) evidence increased its annotated disorder content from 15.9% to 27.1%. Users that want to exclude specific evidence types can easily filter them from the entry interface and the feature viewer gets updated accordingly.

As AlphaFold2 produces structural models for complete proteins, it is possible to compare disorder content based on experimental annotation and AlphaFold2 pLDDT scores. AlphaFoldDB (23) categorizes structure predictions at the residue level into confidence levels of ‘very high’, ‘confident’, ‘low’ and ‘very low’ based on the pLDDT score provided as output by the AlphaFold2 software (1). Predictions with a low confidence level (pLDDT < 70) are predominantly unstructured and often exhibit limited secondary structure elements amidst random coil structures.

**Figure 1.**
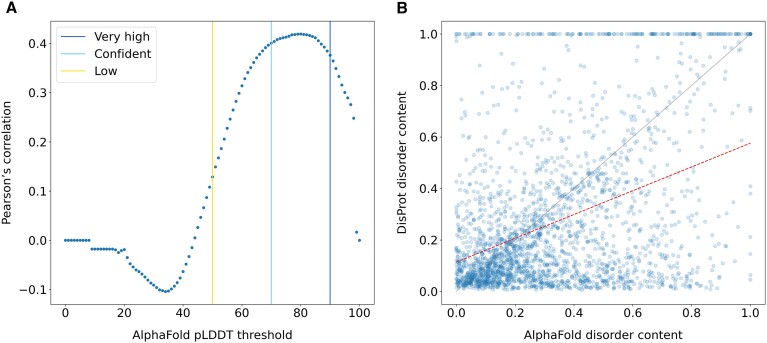
Comparison of the disorder content at the protein level in DisProt and AlphaFoldDB. The disorder content is calculated as the fraction of disordered residues over the protein sequence length. DisProt disorder content corresponds to the fraction of residues in the consensus, which includes structurally disordered regions. Only DisProt proteins with an AlphaFold structure covering the entire protein sequence in AlphaFoldDB were considered, *n* = 2356. (**A**) Correlation of the disorder content between DisProt and AlphaFold when different pLDDT thresholds are selected. (**B**) Comparison of the disorder content between DisProt and AlphaFold when the AlphaFold pLDDT < 70. The red dotted line represents the linear least-squares regression between the two dimensions, with slope 0.462 ± 0.021 and intercept 0.114 ± 0.009.

In Figure 1A, we show the correlation of the disorder content, i.e. the fraction of disordered residues in the protein sequence, between DisProt and AlphaFold2 when different pLDDT thresholds are selected. DisProt and AlphaFold2 correlate well when the pLDDT threshold is between 70 and 90 with a maximum correlation at pLDDT = 80 (Pearson's correlation 0.42, *P*-value 6.26e^−101^). Despite a good correlation between these two alternative descriptions of disorder, they show some important differences.

In Figure 1B, we plotted the disorder content for each protein in DisProt and AlphaFold2 (pLDDT < 70). A number of proteins are annotated with high disorder content in DisProt and at the same time predicted with high confidence in AlphaFoldDB (Figure 1B, above the diagonal). These cases correspond to proteins proven experimentally to be disordered in isolation but that fold upon binding. For example, *Antitoxin YefM* (DP01488, P69346) folds upon protein binding, *Calsequestrin-1* (DP00132, P07221) folds upon Ca^2+^ ion binding and the *Eukaryotic translation initiation factor 4E-binding protein 2* (DP01293, Q13542) folds upon phosphorylation. The AlphaFold2 method learned the folded version of these proteins from similar examples available in well-structured PDB complexes, or simply it was trained to prioritize structured solutions. The existence of low complexity regions within IDRs could also be responsible for some of these cases (24,25). Below the diagonal of Figure 1B, there are cases for which AphaFold predicts most of the structure with a low pLDDT but the corresponding region in the protein sequence is not annotated as disordered in DisProt. Assuming a low pLDDT is a good proxy for disorder prediction, those proteins are potential cases of under annotation in DisProt and worth to be inspected for additional curation. Indeed, most biophysical studies in the literature focus on a single region instead of the full protein and therefore it is difficult for a curator to evaluate that the collected evidence is sufficient to represent the structural properties of the entire protein. An alternative explanation for the low AlphaFold confidence, in addition to the lack of a good template for homology modeling, could be the low quality of the Multiple Sequence Alignment (MSA) input. Shallow MSAs derived from protein families with a limited conservation at the species level have been shown to decrease the confidence of the AlphaFold prediction and increase the structural diversity in the top ranking solutions (26). Examples of proteins with AlphaFold low confidence predictions but low content of annotated disorder in DisProt include the *Nuclear fragile X mental retardation-interacting protein 1* (DP03731, Q9UHK0) and *Protein AF-10* (DP02852, P55197). The predictions of disorder provided by the CAID Prediction Portal (27) for these proteins suggest that they are mostly disordered. Additional examples include all collagen alpha proteins, like the *Collagen alpha-4(IV) chain* (DP03072, P53420), which are currently under-annotated in DisProt.

### Disorder function

One of the most relevant advancements of DisProt in recent years was the increase of functional annotations both in terms of volume and diversity. The importance of DisProt in capturing disorder related function is recognized by other core data resources so that DisProt, after joining the Gene Ontology Consortium and since DisProt release 2022_03, is able to propagate functional annotation to other core data resources, such as UniProtKB. For example, ‘negative regulation of cell population proliferation’ (GO:0008285) function of the *von Hippel-Lindau disease tumor suppressor* protein (UniProtKB P40337) is provided by DisProt along with 9 other GO terms.

**Figure 2.**
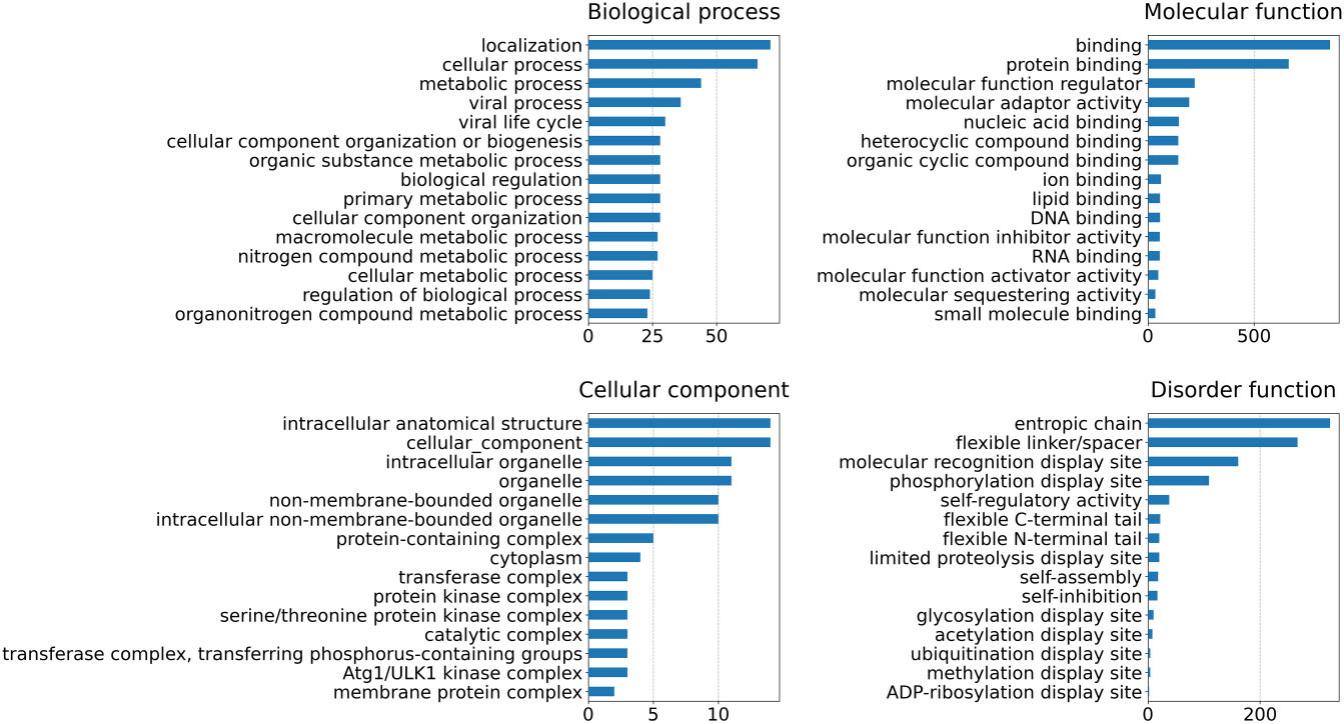
The number of DisProt proteins annotated with functional terms. The statistic is provided for the three Gene Ontology namespaces, as well as for the ‘Disorder function’ aspect from the IDPO ontology. The calculation considers only the first 15 most used annotation terms. Before the calculation, both GO and IDPO terms were propagated to the corresponding ontology root. Proteins with multiple identical annotations, e.g. when different articles report the same experimental evidence, are counted only once.

The adoption of GO in addition to the IDPO ontology to annotate the function of disordered proteins and regions let us to expand the repertoire of captured functions and at the same time to be more precise. The total number of different terms used in the current release as well as the number of annotated proteins and regions, is reported in Table 3. In Figure 2, we reported the number of proteins annotated with functional terms for the most used terms.

**Table 3.** Function and structural annotation in DisProt for each ontology aspect

Ontology	Aspect	Terms	Proteins	Regions
GO	Biological process	415	157	320
GO	Cellular component	36	17	37
GO	Molecular function	165	980	2879
IDPO	Disorder function	20	491	735
IDPO	Structural state	5	2648	6004
IDPO	Structural transition	10	510	847

The number of terms is the number of unique terms after propagating each term to its ontology root.

Regarding the GO aspects, terms from the molecular function branch are the most frequently used. The binding function is the most abundant, followed by regulation activity. This result, taken together with the most abundant biological process function, ‘localization’, confirms the predominant role of IDPs in cell regulation processes. Instead, it is difficult to draw any conclusions from the cellular component aspect due to the limited number of annotated proteins. Regarding the ‘Disorder function’ aspect of the IDPO, it is clear how DisProt mainly annotates linkers and molecular recognition display sites. The latter confirms the predominant role of ID in regulation processes. Finally, it is worth noting that this statistic does not provide a complete overview of the functions of disordered proteins but rather reflects the information available in the database and could be biased by the specific thematic datasets and organisms currently available. To obtain a comprehensive analysis of the functions of these proteins, it is necessary to integrate the function annotations from other databases, such as UniProtKB.

### Implementation

Compared to the previous publication, our focus has been on enhancing data consistency and reliability. This involved integrating MIADE, new GO, and ECO terms. Additionally, we have made significant improvements to the DisProt web interface, particularly the FeatureViewer (28) available on the entry page. The updated FeatureViewer includes several new tracks to provide valuable insights. First, the InterPro (29) features track highlights conserved domains according to the Pfam definition (30) and disordered regions, as provided by MobiDB-lite (31). Another track specifically highlights disordered regions derived from PDB missing residues, as calculated by MobiDB (consensus track). In addition to these enhancements, we have integrated a track that reports the AlphaFold2 confidence at the residue level. This information is divided into four different confidence intervals corresponding to different colors, as reported in AlphaFoldDB.

Overall, the upgraded FeatureViewer greatly improves the understanding and analysis of functional elements within the protein sequence. It is particularly beneficial for biocurators, allowing them to focus on protein regions that are more likely to be disordered or areas that have received less research attention.An interactive annotation form is also now accessible for external contributors, enabling them to submit new annotations missing in DisProt. The submission form allows annotations about the structural state or function of an IDP or IDR through the identification of a bibliographic source (PubMed ID), and the selection of the relevant ECO term. The form is compliant with MIADE and allows users to optionally provide additional details such as their name, email address, ORCID identifier, and comments.

### Training material

One of the central activities in DisProt involves meticulously annotating IDRs from pertinent research articles. The curation activity in DisProt entails thorough evaluation, collection and integration of experimental data. To join the DisProt curators team, it is essential to attend our specialized courses to acquire a good understanding of the curation process. Training courses are available on the ELIXIR eLearning platform (https://elixir.mf.uni-lj.si/), offered both in English and Spanish languages. These courses aim to provide comprehensive guidelines for biocurators, covering all aspects of IDP biology, curation processes, structural and functional annotations and submission procedures.

Moreover, in order to assist users in acquiring the necessary knowledge to navigate the DisProt database, explore its primary features, and interpret data it contains, two webinars are accessible on the ELIXIR training Portal (TESS) (https://tess.elixir-europe.org/). One webinar serves as an introductory guide to the DisProt website, covering diverse sections (https://tess.elixir-europe.org/materials/an-introduction-to-disprot). The second webinar is deeper into data interpretation and explores the various ways users can leverage the data for their scientific research (https://tess.elixir-europe.org/materials/exploring-structural-and-functional-annotations-of-idps-with-disprot).

## Conclusions

DisProt is a comprehensive database that systematically collects and standardizes experimental evidence on protein disorder extracted from scientific literature using a rigorous protocol and established standards. The database has been experiencing remarkable growth, expanding its content by 30% every 2 years. This progress has been made possible by the active involvement of a dedicated community of biocurators with a keen interest in molecular biology and biophysical methods. In recognition of their contributions and to foster engagement, DisProt was the pioneering database to integrate gamification concepts through its connection with the APICURON service (10).

While maintaining a steady growth rate, DisProt has recently achieved significant advancements, notably a shift in curation practices that has enhanced precision through the adoption of MIADE guidelines (20). This paradigm shift has also broadened the scope of functional annotations by incorporating Gene Ontology (GO) terms. In fact, DisProt has become a member of the Gene Ontology Consortium (12), ensuring the automatic propagation of its annotations into UniProtKB. The rapid expansion of functional annotations has resulted in a twofold increase over the past four years. This growth sets the stage for the development of next-generation, highly accurate methods for predicting the functions of intrinsically disordered proteins (IDPs). DisProt data have also been used to organize a sub-challenge in CAFA4.

Another notable improvement in DisProt is the adoption of additional Evidence and Conclusion Ontology (ECO) terms (22). This update has relaxed previous curation constraints, enabling the capture of evidence that was previously overlooked, including statements from authors. The improved coverage at the residue level for bona fide disordered sites has enhanced the correlation with disorder prediction methods, as evidenced by recent results from CAID, where DisProt serves as a trusted benchmark (7,11). DisProt disorder has also been recently integrated into InterPro (29).

The long-term sustainability of DisProt is ensured by its central role in various initiatives involving large communities of bioinformaticians dedicated to studying protein disorder. Notable examples include the ML4NGP COST Action and the ELIXIR IDP Community, both of which foster collaboration and knowledge exchange among experts in the field.

Overall, DisProt continues to evolve as a vital resource for the scientific community, providing standardized and curated experimental evidence on protein disorder and driving advancements in the study of IDPs.

## Data Availability

DisProt is freely available at https://disprot.org.

## Appendix

### DisProt Consortium

Vasileios Sagris^3^, Vasilis J Promponas^3^, Anastasia Chasapi^4^, Erzsébet Fichó^5^, Galo E Balatti^6,7^, Gustavo Parisi^6,7^, Martín González Buitrón^6,7^, Gabor Erdos^8^, Matyas Pajkos^8^, Zsuzsanna Dosztányi^8^, Laszlo Dobson^9,10^, Alessio Del Conte^1^, Damiano Clementel^1^, Edoardo Salladini^1^, Emanuela Leonardi^1^, Fatemeh Kordevani^1^, Hamidreza Ghafouri^1^, Luiggi G Tenorio Ku^1^, Alexander Miguel Monzon^11^, Carlo Ferrari^11^, Zsófia Kálmán^12,10^, Juliet F Nilsson^13^, Jaime Santos^14,15^, Carlos Pintado-Grima^14^, Salvador Ventura^14^, Veronika Ács^16^, Rita Pancsa^16^, Mariane Goncalves Kulik^17^, Miguel A Andrade-Navarro^17^, Pedro José Barbosa Pereira^18^, Sonia Longhi^19^, Philippe Le Mercier^20^, Julian Bergier^21^, Peter Tompa^16,22,23^, Tamas Lazar^22,23^

^3^Bioinformatics Research Laboratory, Department of Biological Sciences, University of Cyprus, Nicosia, Cyprus

^4^Biological Computation & Process Laboratory (BCPL), Chemical Process & Energy Resources Institute, Centre for Research & Technology Hellas (CERTH), Thessaloniki, Greece

^5^Cytocast Hungary Kft., Szervita sqr. 8, Budapest H-1052, Hungary

^6^Departamento de Ciencia y Tecnología, Universidad Nacional de Quilmes, Buenos Aires, Argentina

^7^Consejo Nacional de Investigaciones Científicas y Técnicas (CONICET), Buenos Aires, Argentina

^8^Department of Biochemistry, Eötvös Loránd University, Pázmány Péter stny 1/c, Budapest H-1117, Hungary

^9^Department of Bioinformatics, Semmelweis University, Tűzoltó u. 7, Budapest, 1094, Hungary

^10^Structural and Computational Biology Unit, European Molecular Biology Laboratory, Meyerhofstraße 1, Heidelberg, 69117, Germany

^11^Department of Information Engineering, University of Padova, Padova, Italy

^12^Faculty of Information Technology and Bionics, Pázmány Péter Catholic University, Práter u. 50/A, Budapest, 1083 Hungary

^13^INRAE, Aix Marseille University, UMR1163 Biodiversité et Biotechnologie Fongiques, 13009 Marseille, France

^14^Institut de Biotecnologia i de Biomedicina (IBB) and Departament de Bioquímica i Biologia Molecular, Universitat Autònoma de Barcelona, 08193 Bellaterra, Barcelona, Spain

^15^Center for Molecular Biology of Heidelberg University (ZMBH), Heidelberg, Germany

^16^Institute of Enzymology, HUN-REN Research Centre for Natural Sciences, Budapest H-1117, Hungary

^17^Institute of Organismic and Molecular Evolution, Faculty of Biology, Johannes Gutenberg University of Mainz, Germany

^18^Instituto de Investigação e Inovação em Saúde, Universidade do Porto, 4200–135 Porto, Portugal

^19^Lab. Architecture et Fonction des Macromolécules Biologiques (AFMB), UMR 7257, Aix Marseille University and Centre National de la Recherche Scientifique (CNRS), 163 Avenue de Luminy, Case 932, 13288, Marseille, France

^20^Laboratoire des Lyssavirus, Institut Pasteur, 75724 Paris Cédex 15, France

^21^Laboratorio de Ingeniería Genética y Biología Celular y Molecular, Área Virosis de Insectos, Instituto de Microbiología Básica y Aplicada, Departamento de Ciencia y Tecnología, Universidad Nacional de Quilmes, Buenos Aires, Argentina

^22^VIB-VUB Center for Structural Biology, Vlaams Instituut voor Biotechnologie (VIB), Brussels, Belgium

^23^Structural Biology Brussels, Department of Bioengineering, Vrije Universiteit Brussel, Brussels, Belgium
