# Correction to ‘DisProt in 2024: improving function annotation of intrinsically disordered proteins’

**DOI:** 10.1093/nar/gkaf228

**Published:** 2025-03-13

This is a correction to: Maria Cristina Aspromonte (*et al.*), DisProt in 2024: improving function annotation of intrinsically disordered proteins, *Nucleic Acids Research*, Volume 52, Issue D1, 5 January 2024, Pages D434–D441, https://doi.org/10.1093/nar/gkad928

In the originally published version of the manuscript there was an error in the fourth author's last name. This should read: “Bouhraoua” instead of: “Bouharoua”.

The error is outlined only in this notice to preserve the version of record.

